# Metformin protects against myocardial ischemia-reperfusion injury and cell pyroptosis via AMPK/NLRP3 inflammasome pathway

**DOI:** 10.18632/aging.202143

**Published:** 2020-11-24

**Authors:** Jing Zhang, Lelin Huang, Xing Shi, Liu Yang, Fuzhou Hua, Jianyong Ma, Wengen Zhu, Xiao Liu, Rui Xuan, Yunfeng Shen, Jianping Liu, Xiaoyang Lai, Peng Yu

**Affiliations:** 1 Department of Anesthesiology, The Second Affiliated Hospital of Nanchang University, Jiangxi 3300063, Nanchang, China; 2 Department of Anesthesiology, Lushan Rehabilitation and Recuperation Center, PLA Joint Service Forces, Jiujiang 3320000, China; 3 Department of Metabolism and Endocrinology, The Second Affiliated Hospital of Nanchang University, Jiangxi 330006, Nanchang, China; 4 Department of Pharmacology and Systems Physiology, University of Cincinnati College of Medicine, Cincinnati, OH 45267, USA; 5 Department of Cardiology, The First Affiliated Hospital of Sun Yat-Sen University, Guangzhou 510080, Guangdong, China; 6 Department of Cardiology, The Second Affiliated Hospital of Sun Yat-Sen University, Guangzhou 510080, Guangdong, China

**Keywords:** metformin, pyroptosis, myocardial ischemia reperfusion injury, APMK, NLRP3

## Abstract

Ischemia/reperfusion (I/R) injury is a life-threatening vascular emergency following myocardial infarction. Our previous study showed cardioprotective effects of metformin against myocardial I/R injury. In this study, we further examined the involvement of AMPK mediated activation of NLRP3 inflammasome in this cardioprotective effect of metformin. Myocardial I/R injury was simulated in a rat heart Langendorff model and neonatal rat ventricle myocytes (NRVMs) were subjected to hypoxi/reoxygenation (H/R) to establish an in vitro model. Outcome measures included myocardial infarct size, hemodynamic monitoring, myocardial tissue injury, myocardial apoptotic index and the inflammatory response. myocardial infarct size and cardiac enzyme activities. First, we found that metformin postconditioning can not only significantly alleviated myocardial infarct size, attenuated cell apoptosis, and inhibited myocardial fibrosis. Furthermore, metformin activated phosphorylated AMPK, decreased pro-inflammatory cytokines, TNF-α, IL-6 and IL-1β, and decreased NLRP3 inflammasome activation. In isolated NRVMs metformin increased cellular viability, decreased LDH activity and inhibited cellular apoptosis and inflammation. Importantly, inhibition of AMPK phosphorylation by Compound C (CC) resulted in decreased survival of cardiomyocytes mainly by inducing the release of inflammatory cytokines and increasing NLRP3 inflammasome activation. Finally, in vitro studies revealed that the NLRP3 activator nigericin abolished the anti-inflammatory effects of metformin in NRVMs, but it had little effect on AMPK phosphorylation. Collectively, our study confirmed that metformin exerts cardioprotective effects by regulating myocardial I/R injury-induced inflammatory response, which was largely dependent on the enhancement of the AMPK pathway, thereby suppressing NLRP3 inflammasome activation.

## INTRODUCTION

Cardiovascular diseases, including acute myocardial infarction, are global leading cause of death [1, 2]. Currently, rapid and safe restoration of blood supply to ischemic myocardium is considered the best and the most effective treatment for acute myocardial infarction [3]. However, revascularization may also aggravate myocardial damage, produce a second blow to the myocardium, and cause myocardial reperfusion injury after ischemia [4]. Exploring prevention measures and action mechanisms of ischemia/reperfusion (I/R) injury is of crucial research significance for improving the prognosis and survival rate of patients with cardiovascular disease. Simple ischemic pre-conditioning and post-conditioning usually affect and block the vascular system, thus causing different degrees of physical damage. So far, several drugs have been shown to have a cardioprotective effect, preventing adverse effects of ischemic treatment on blood vessels [5]. Because of the unpredictability of ischemia, pharmacological postconditioning after ischemia-reperfusion has shown to be convenient and feasible. Our previous research revealed that metformin postconditioning could protect against myocardial I/R injury [6, 7].

Metformin is widely used for the treatment of type 2 diabetes and metabolic syndrome due to its strong ability to enhance insulin sensitivity and its safety [8]. Previous studies have demonstrated that metformin can inhibit I/R injury through multiple signaling pathways in organs such as intestines, kidneys, heart, and brain [9–12]. Moreover, the anti-inflammatory properties of metformin have been described in several models of autoimmune inflammation, such as arthritis, uveitis, and hepatitis [13–15]. For example, a recent study showed that metformin effectively improves intestinal I/R injury by inhibiting pyroptosis through an adenosine monophosphate-activated protein kinase (AMPK) [16]. Similarly, multiple drugs exert the effect of resisting pyroptosis by activating AMPK.

Pyroptosis is a newly discovered programmed cell death process, which frequently occurs in stressful environments of various organs and tissues [17]. Pyroptosis can also induce immoderate cell inflammatory damage [18]. In addition to apoptosis, necrosis, and autophagy, pyroptosis has a crucial role in myocardial I/R injury [19]. NOD-like receptor protein 3 (NOD-like receptor protein 3, NLRP3) inflammasome, as a participant of the inflammatory immune response, is closely related to cardiovascular diseases [20]. Numerous studies have confirmed that NLRP3 inflammasome is involved in the occurrence and development of myocardial I/R injury, cardiomyopathy, arrhythmia, and other diseases [21–23]. However, there are no reports on the role of NLRP3 inflammasome activation in myocardial I/R injury and metformin-related intervention studies.

In this study, we investigated the cardioprotective effect of metformin against myocardial I/R injury through inhibition of NLRP3 inflammasome and activation of AMPK. Metformin post-conditioning in animal tissue and cell models was used to investigate the effect and mechanism of metformin-mediated AMPK activation on intracellular NLRP3 inflammasome when resisting myocardial I/R injury. Compared with drug pre-conditioning, drug post-conditioning is easier to implement in clinic. Moreover, drug post-conditioning is an important measure to prevent myocardial I/R injury.

## RESULTS

### Metformin reduces myocardial infarction size, suppresses myocardial tissue enzyme content and improves hemodynamic performance

To confirm the cardioprotective effect of metformin postconditioning on myocardial I/R injury, myocardial infarction size was measured by using 1%TTC. Consistent with our previous studies [6, 24, 25], I/R injury increased myocardial infarction size in I/R group (*P* < 0.05). Compared with the I/R group, myocardial infarction size was decreased in the MET group (*P* < 0.05).

**Figure 1:**
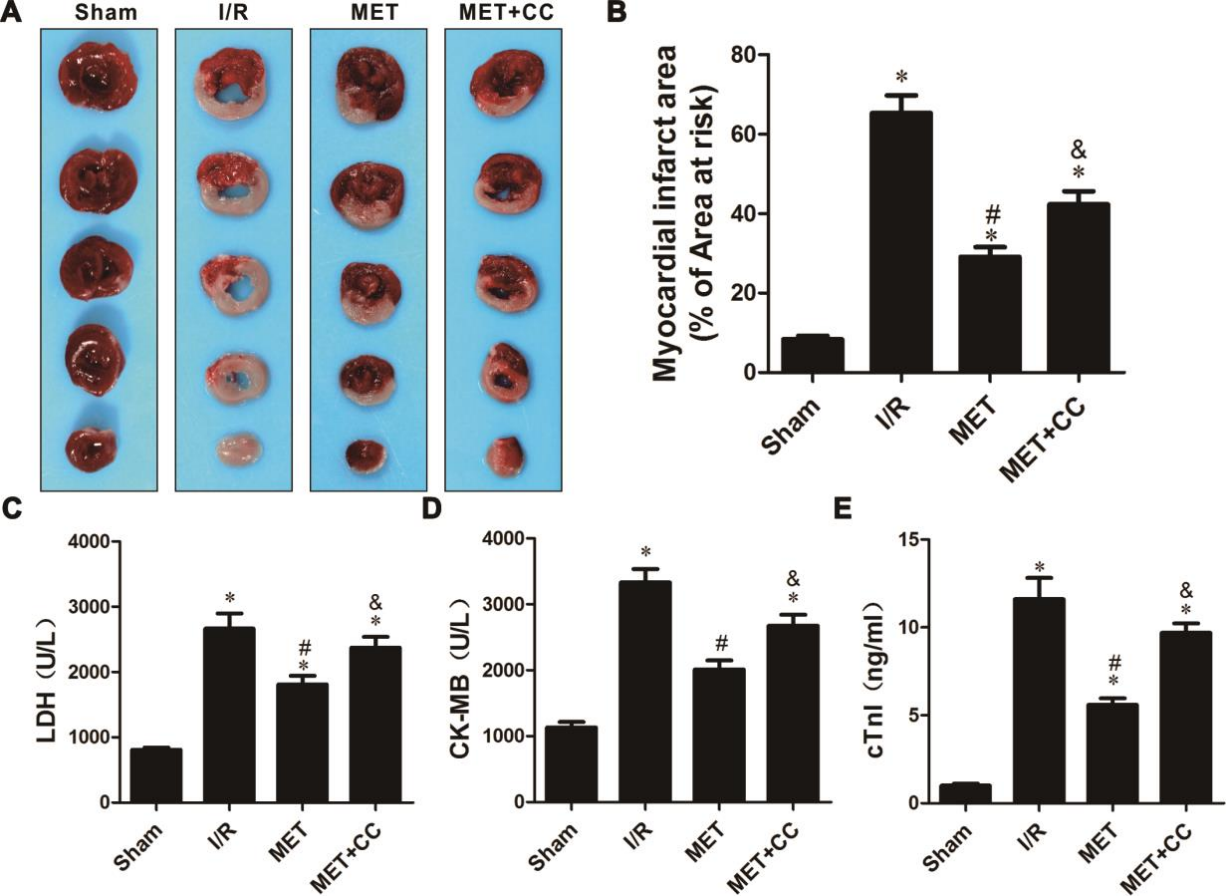
**Metformin ameliorated IR-induced cardiac tissue damage in a rat I/R injury model.** (**A**) Representative images of myocardial infarct size stained by TTC staining. (**B**) Myocardial infarct volumes presented as percentage of infarct area/area at risk (n = 6–8 per group). (**C**) Mean levels of lactate dehydrogenase (LDH) in all groups (n = 6 per group). (**D**) Mean levels of creatine kinase-MB (CK-MB) in all groups (n = 6 per group). (**E**) Mean levels of cardiac troponin I (cTnI) in all groups (n = 6 per group). Values are expressed as the mean ± SEM. * *P* < 0.05 vs. Sham. # *P* < 0.05 vs. I/R. & *P* < 0.05 vs. MET.

Next, we examined the effect of metformin postconditioning on the activation of AMPK. Isolated hearts were treated with Compound C (CC), a small molecule compound functioning as a direct inhibitor of AMPK, in addition to metformin. As shown in Figure 1A, 1B, myocardial infarction size was increased in the MET+CC group compared to hearts treated with metformin alone (*P* < 0.05). Furthermore, the contents of myocardial tissue enzyme LDH, CK-MB, and cTnl (*P* < 0.05, Figure 1C–1E) were up-regulated after the I/R injury, but down-regulated after using metformin (P < 0.05, Figure 1C–1E). Compared with the MET group, myocardial tissue enzyme LDH, CK-MB, and cTnl were increased in the MET+CC group (*P* < 0.05, Figure 1C–1E).

To further understand the role that metformin-induced AMPK activation has in the protection of cardiac function, the hemodynamics indexes in all groups were monitored and recorded at T0, T1, T2, T3, and T4. The hemodynamics indexes at T0 in all groups were significantly different (*P* > 0.05, Table 1). Compared with T0, HR and LVSP were decreased, while LVEDP was increased at T1, T2, T3, and T4 in the I/R, MET, and MET+CC groups (*P* < 0.05, Table 1). Compared with the Sham group, HR and LVSP were also decreased, while LVEDP was increased at T1, T2, T3, T4 in the other groups (*P* < 0.05, Table 1). As expected, metformin postconditioning significantly inhibited the changes caused by myocardial I/R injury in the MET group (P < 0.05, Table 1). However, compared with the MET group, decreased HR, LVSP, and increased LVEDP were observed in the MET+CC group (*P* < 0.05, Table 1). To sum up, these results indicated that AMPK is associated with reduced I/R injury-induced myocardial infarction and cardiac dysfunction caused by metformin post-conditioning.

**Table 1:** Hemodynamics in vitro experiments.

	**Group**	**T_0_**	**T_1_**	**T_2_**	**T_3_**	**T_4_**
HR (min^-1^)	Sham	267 ± 21	273 ± 19	302 ± 23	298 ± 20	331 ± 31
	I/R	298 ± 19	220 ± 22^*#^	201 ± 17^*#^	183 ± 30^*#^	156± 32^*#^
	MET	303 ± 20	245 ± 23^*#&^	260± 17^*#&^	231 ± 25^*#&^	209 ± 20^*#&^
	MET+CC	309 ± 25	224 ± 20^*#^	209 ± 25^*#†^	179 ± 15^*#†^	147± 28^*#†^
LVSP (mmHg)	Sham	103 ± 7	113 ± 6	110 ± 8	109 ± 8	115 ± 11
	I/R	108 ± 7	93 ± 7^*#^	63 ± 9^*#^	51 ± 7^*#^	44 ± 6^*#^
	MET	111 ± 11	101 ± 9^#&^	84 ± 10^*#&^	71 ± 13^*#&^	61± 9^*#&^
	MET+CC	112 ± 13	89 ± 7^*#†^	61 ± 7^*#†^	53 ± 8^*#†^	46± 7^*#†^
LVEDP (mmHg)	Sham	7.8 ± 0.9	7.4 ± 0.7	8.2 ± 1.3	7.9 ± 0.9	8.3 ± 1.1
	I/R	8.4 ± 1.2	27.6 ± 3.9^*#^	39.7 ± 9.4^*#^	51.8 ± 5.9^*#^	73.3 ± 8.0^*#^
	MET	7.3 ± 0.8	13.5 ± 2.9^*#&^	25.5 ± 7.5^*#&^	40.6 ± 5.5^*#&^	53.9 ± 8.6^*#&^
	MET+CC	7.1 ± 0.7	24.9 ± 6.3^*#†^	41.9 ± 7.1^*#†^	53.3 ± 7.9^*#^	71.5 ± 6.1^*#†^

* *P* < 0.05 vs T0; # *P* < 0.05 vs SHAM group; & *P* < 0.05 vs IR group; ^†^ *P* < 0.05 vs IR group. Values are means ± standard deviation. n = 6 /group. HR, heart rate; LVSP, left ventricular peak pressure; LVEDP, left ventricular end diastolic pressure. T_0_, equilibration; T_1_, 30 min after reperfusion; T_2_, 60 min after reperfusion; T_3_, 90 min after reperfusion; T_4_, 2h after reperfusion. SHAM, sham control; IR, ischemia reperfusion; MET, metformin postconditioning; CC, compound C, the metformin inhibitor.

### Metformin alleviates the degree of cardiac cell damage and fibrosis by reducing the expression of Col-I and Col-III

**Figure 2:**
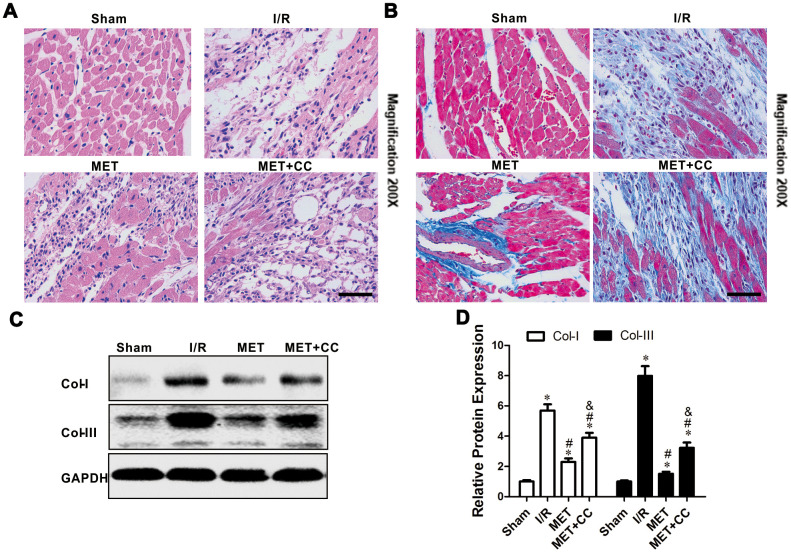
**Metformin protected I/R injury-induced myocardial injury by suppressing collagen synthesis.** (**A**) Representative pictures of H&E-stained cardiac sections and (**B**) representative images of myocardial fibrosis stained with the Masson trichrome method (n = 4 per group). Magnification 200x, Scale bar = 100μm; (**C**) Collagen-related proteins in the ischemic area, including COL-I, COL-III and GAPDH were examined by Western blot analysis. (**D**) Quantitative analysis of COL-I and COL-III expression (n = 4 per group). Values are expressed as the mean ± SEM. * *P* < 0.05 vs. Sham. # *P* < 0.05 vs. I/R. & *P* < 0.05 vs. MET.

To further examine the cardioprotective effect of metformin postconditioning, the degree of damaged myocardium and fibrotic scar tissue was determined. As shown in Figure 2A, HE staining indicated that the structure of myocardial tissue in the Sham group was clear; the cardiomyocytes were normally arranged, and there was no cardiomyocytes necrosis, inflammatory cell infiltration, and fibrous hyperplasia. In the I/R group, the cardiomyocytes arrangement was messy, and the cardiomyocytes were enlarged. Among the cardiomyocytes, small blood vessels were dilated and congested, and a large number of inflammatory cells and fibrous hyperplasia were found (Figure 2A). Compared with the I/R group, the MET group had a clear myocardial tissue structure and lighter myocardial damage. Also, a small number of inflammatory cells in the myocardial interstitium, and a buildup of collagen fibers between the myocardium, during which the small blood vessels dilated and the congestion was lighter, were detected (Figure 2A). In the MET+CC group, the myocardial tissue was aggravated compared to the MET group but reduced compared to the I/R group (Figure 2A).

Mason staining was further used to detect myocardial collagen deposition in all groups. Only a small amount of collagen was found in the myocardial interstitium in the Sham group (Figure 2B). Collagen deposition in I/R group significantly increased compared with the Sham group (Figure 2B). Compared with I/R group, the collagen fibers accumulation between cardiomyocytes was significantly reduced in the MET group (Figure 2B). In comparison, the positive staining of myocardial collagen in the MET+CC group was significantly higher than that in the MET group (Figure 2B). Collagen type I (Col-I) and collagen type III (Col-III) have an important role in the entire collagen network of myocardial tissue and can reflect the degree of myocardial fibrosis.

Next, we examined whether metformin down-regulates collagen synthesis through AMPK activation. Compared with the Sham group, the expression levels of Col-I and Col-III were up-regulated in other groups (*P* < 0.05, Figure 2C, 2D). Moreover, metformin administration down-regulated the expression levels of Col-I and Col-III (*P* < 0.05, Figure 2C, 2D). Compared with the MET group, the expression levels of Col-I and Col-III were significantly up-regulated in the MET+CC group (*P* < 0.05, Figure 2C, 2D). Taken together, these data indicate that metformin substantially reduces histopathological necrotic areas and improves cardiac fibrosis.

### Metformin restrains apoptotic cardiomyocytes and apoptosis-related protein expression level involved by activating the AMPK pathway

**Figure 3:**
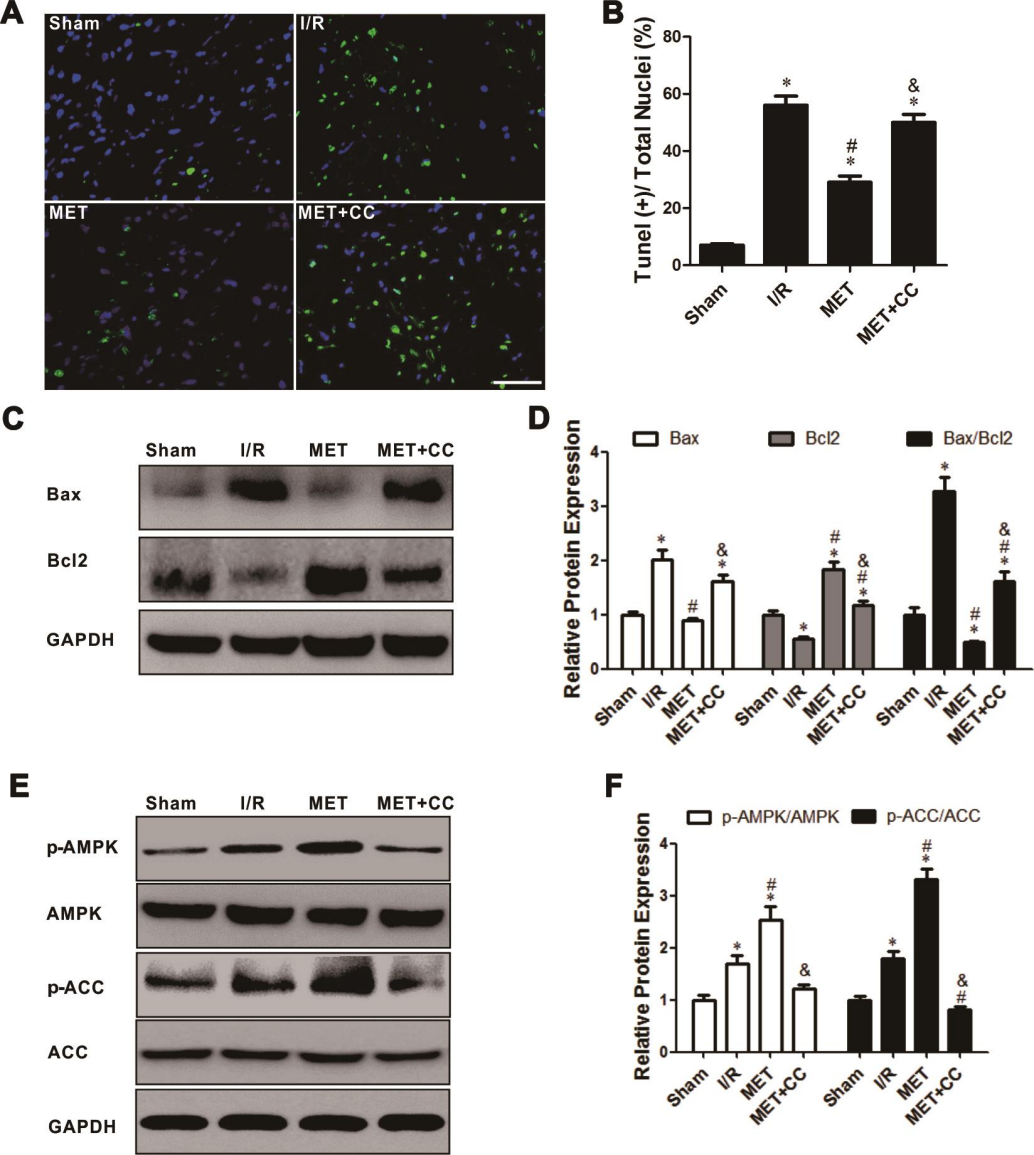
**Activation of AMPK with Metformin protected against myocardial I/R injury induced apoptosis.** (**A**) Top representative TUNEL-stained (green fluorescence) and DAPI-stained (blue fluorescence) photomicrographs are shown (Magnification 200x, Scale bar = 50 μm). (**B**) Bar graph represents the quantification of apoptotic cells (green fluorescence)/the total number of nucleated cells (blue fluorescence, n = 6 per group). (**C**) Apoptosis-related proteins in the ischemic area, including Bax, Bcl2 and GAPDH were examined by Western blot analysis. (**D**) Quantitative analysis of Bax, Bcl2 and calculate the ratio of Bax/Bcl2 (n = 4 per group). (**E**) AMPK pathway-related proteins in the ischemic area, including p-AMPK, AMPK, p-ACC, ACC and GAPDH were examined by Western blot analysis. (**F**) Quantitative analysis of p-AMPK, AMPK, p-ACC, ACC expression and calculate the ratio of p-AMPK/AMPK and p-ACC/ACC (n = 4 per group). Values are expressed as the mean ± SEM. * *P* < 0.05 vs. Sham. # *P* < 0.05 vs. I/R. & *P* < 0.05 vs. MET.

Apoptosis of myocardial cells has a crucial part in I/R injury [26]. In this study, we measured apoptosis cells using TUNEL staining. Our results showed that the green fluorescence of myocardial tissue in the IR group was significantly higher than in the Sham group (*P* < 0.05, Figure 3A, 3B). After metformin administration, the green fluorescence was significantly reduced (*P* < 0.05, Figure 3A, 3B). These suggested that metformin effectively reduces cardiomyocytes apoptosis. In the MET+CC group, the fluorescence was increased compared with the MET group (*P* < 0.05, Figure 3A, 3B).

Next, the apoptosis-related proteins were analyzed by Western Blot. As shown in Figure 3C, 3D, the expression levels of Bax and Bax/Bcl2 were increased, while the expression of Bcl2 was more decreased in I/R group than in the Sham group (*P* < 0.05); this reaction was reversed in the MET group (*P* < 0.05, Figure 3C, 3D). After the administration of CC, the variation induced by metformin was receded; the expression levels of Bax and Bax/Bcl2 were higher, and the expression level of Bcl2 was lower in the MET+CC group than in the MET group (P < 0.05, Figure 3C, 3D).

As shown in Figure 3E, 3F, the results from Western Blot demonstrated that the expression level ratios of p-AMPK/AMPK and p-ACC/ACC were higher in the MET group than in the I/R group (*P* < 0.05). Moreover, co-administration of CC with metformin largely abolished the activation of the AMPK/ACC axis (*P* < 0.05, Figure 3E, 3F). Thus, our results indicated that the activation of AMPK by metformin lessened IR-injury-induced apoptosis, which is consistent with previous findings [6, 7].

### Metformin suppresses pyroptosis after I/R injury, which is partially depended on AMPK activation

**Figure 4:**
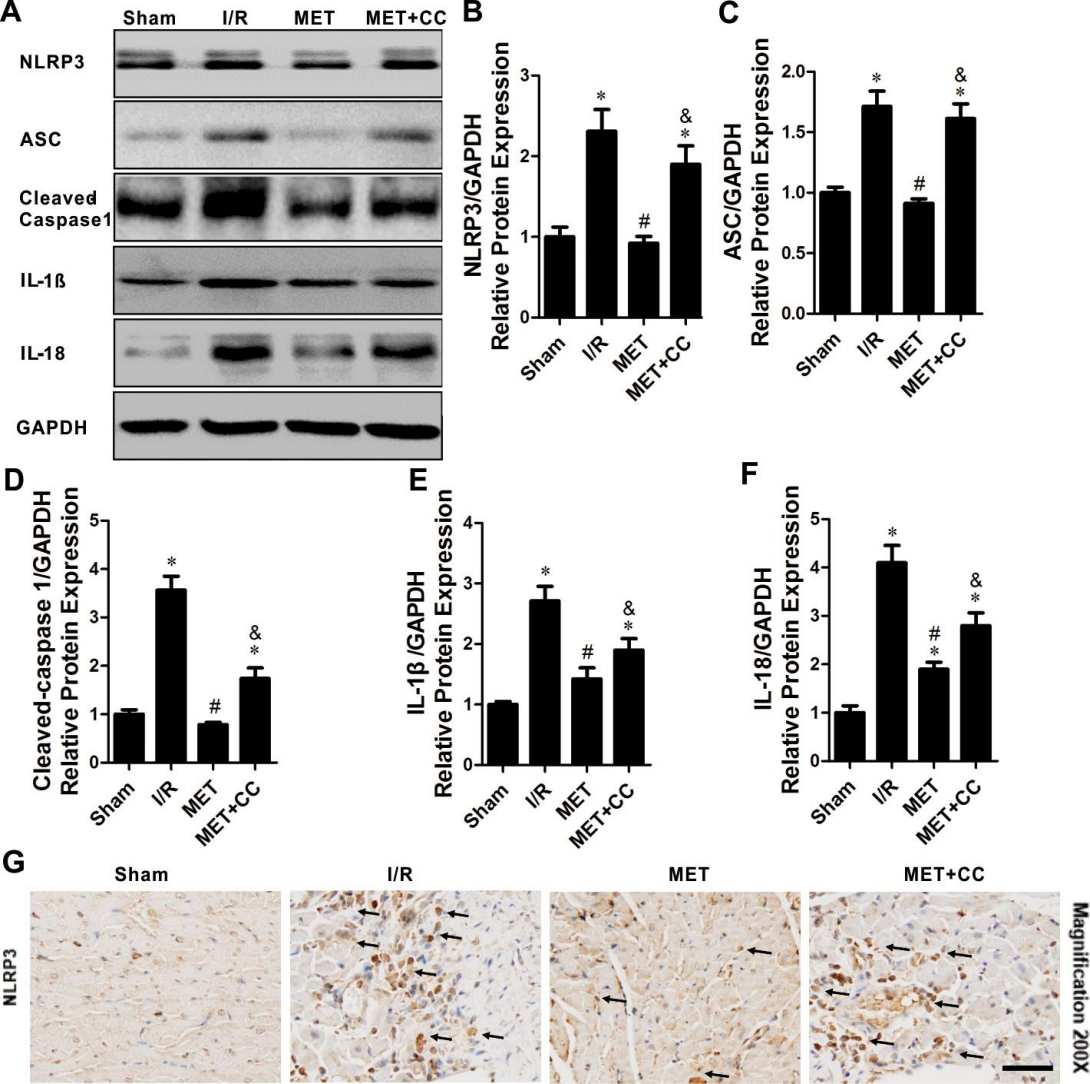
**The inhibiting effects of Metformin on NLRP3 inflammasome activation within the infarct area following myocardial I/R injury.** (**A**) Apoptosis-related proteins in the ischemic area, including NLRP3, ASC, cleaved-caspase 1, IL-1β, IL-18 and GAPDH were examined by Western blot analysis. (**B**–**F**) Quantitative analysis of NLRP3, ASC, cleaved-caspase 1, IL-1β and IL-18 expression (n = 4 per group). (**G**) Bottom representative immunohistochemical-stained NLRP3 in cardiac tissue of each group are shown (n = 4 per group). Magnification 200x, Scale bar = 100 μm; Values are expressed as the mean ± SEM. * *P* < 0.05 vs. Sham. # *P* < 0.05 vs. I/R. & *P* < 0.05 vs. MET.

AMPK has been reported to mediate the activation of NLRP3 inflammasome [21]. Subsequently, we examined whether metformin-mediated AMPK phosphorylation reduces pyroptosis *in vivo.* As shown in Figure 4A–4F, the I/R injury-induced pyroptosis, indicated by elevated expression levels of NLRP3, ASC, cleaved-caspase 1, IL-1β and IL-18, were significantly decreased by metformin (*P* < 0.05). Metformin further inhibited I/R injury-induced activation of NLRP3 inflammasome and its downstream proteins. Moreover, the administration of CC lessened the effect caused by metformin. The expression levels of NLRP3, ASC, cleaved-caspase 1, IL-1β, and IL-18 were higher in the MET+CC group than in the MET group (*P* < 0.05, Figure 4A–4F).

The immunohistochemical staining of myocardium sections of all group hearts revealed the anticipated results. There was no positive staining for NLRP3 in the Sham, while in the I/R injury group, the NLRP3 staining increased (Figure 4G). In myocardium sections of the MET group, staining was decreased compared to the I/R group (Figure 4G). However, compared with the MET group, the NLRP3 staining in MET+CC myocardium sections was markedly increased (Figure 4G). Therefore, these data indicate that metformin leads to AMPK activation and NLRP3 mediated pyroptosis inhibition; this reaction could be reversed by CC treatment.

### Metformin inhibits the release of pro-inflammatory factors by regulating AMPK activation

**Figure 5:**
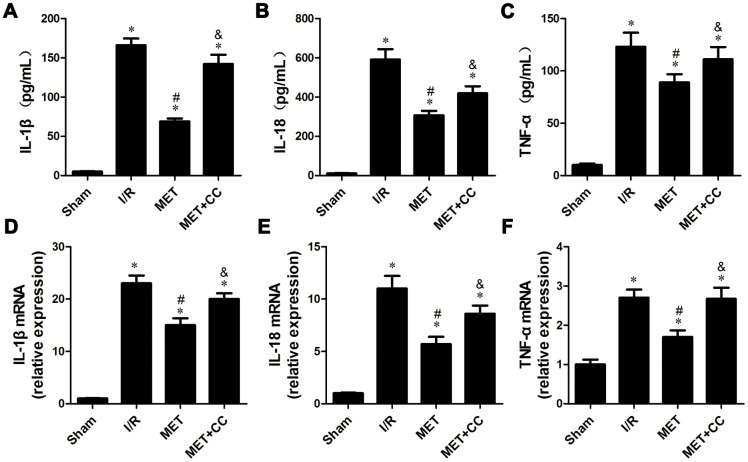
**Metformin inhibited inflammatory cytokines release following myocardial I/R injury.** (**A**) The IL-1β content, (**B**) IL-18 content and (**C**) TNF-α content were detected by ELISA (n = 6–7 per group). (**D**) The mRNA levels of IL-1β, (**E**) IL-18 and (**F**) TNF-α were measured using quantitative RT-PCR (n = 6–7 per group). The housekeeping gene β-actin was used for normalization. Values are expressed as the mean ± SEM. * *P* < 0.05 vs. Sham. # *P* < 0.05 vs. I/R. & *P* < 0.05 vs. MET.

In this experiment, we investigated the effects of metformin on I/R injury-induced release of cytokines associated with inflammation. ELISA estimated the levels of IL-1β, IL-18, and TNF-α in cardiac tissue homogenates. As shown in Figure 5A–5C, the levels of IL-1β, IL-18, and TNF-α were elevated in all groups expect the Sham group (*P* < 0.05). Compared with the I/R group, the levels of IL-1β, IL-18, and TNF-α were reduced in the MET group (*P* < 0.05, Figure 5A–5C), whereas accompanied by metformin and CC, the levels of IL-1β, IL-18, and TNF-α in the MET+CC group were elevated compared with the MET group (*P* < 0.05, Figure 5A–5C).

Next, changes in mRNA expression of IL-1β, IL-18, and TNF-α were examined by RT-PCR. Similar to the above results, we observed that metformin significantly prevented I/R injury-induced the up-regulation of mRNA levels of IL-1β, IL-18, and TNF-α (*P* < 0.05, Figure 5E, 5F). Additionally, the mRNA levels of IL-1β, IL-18, and TNF-α were mildly increased in the MET+CC group compared to that in the single metformin treatment group (*P* < 0.05, Figure 5E, 5F).

### Activation of NLRP3 with nigericin abrogates metformin-induced cardioprotection in NCVMs

**Figure 6:**
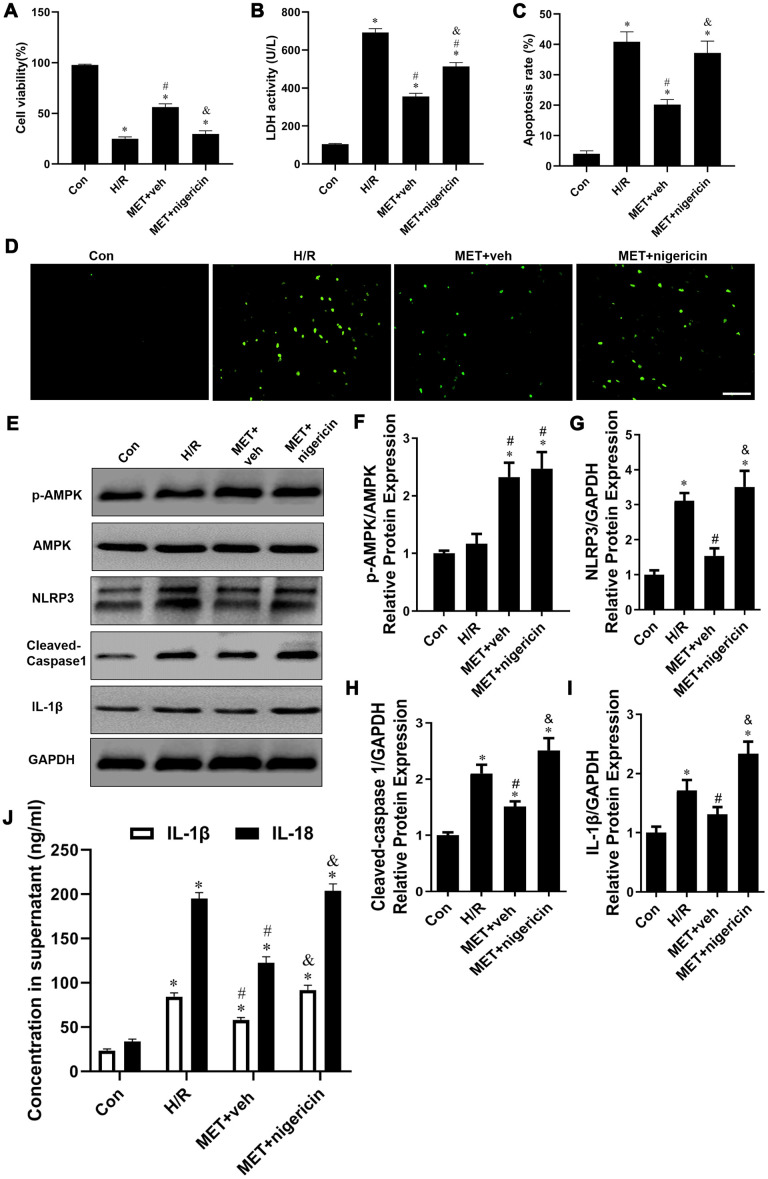
**Activation of NLRP3 with nigericin abolished Metformin-inhibited the release of pro-inflammatory factor *in vitro*.** (**A**) Cell viability was measured by MTT assay (n = 6 per group). (**B**) The supernatant was collected and used to determine the LDH activity (n = 6 per group). (**C**) Statistical results of TUNEL-positive cells per field. (**D**) Tunel assay (apoptotic cells stained in green fluorescence) was performed to assess examine the apoptosis rate of NRVM cells in all groups (n = 6 per group). Magnification 200x, Scale bar = 100 μm; (**E**) Western blots were performed to determine p-AMPK, AMPK, NLRP3, cleaved-caspase 1, IL-1β and GAPDH in the total cell lysates. (**F**–**I**) Quantitative analysis of p-AMPK, AMPK, NLRP3, cleaved-caspase 1, IL-1β expression and calculate the ratio of p-AMPK/AMPK (n = 4 per group). (**J**) The levels of IL-1β, IL-18 and TNF-α in the supernatant from NCVMs were detected by ELISA (n = 6 per group). Values are expressed as the mean ± SEM. * *P* < 0.05 vs. Con. # *P* < 0.05 vs. H/R. & *P* < 0.05 vs. MET+veh.

To further determine the impact of metformin on H/R injury in an NLRP3-dependent way *in vitro*, NCVMs were treated with a special stimulator of NLRP3 (nigericin). We found that the treatment of nigericin reversed the protective effects of metformin. Consistent with our animal data, we observed that metformin substantially increased cell viability (*P* < 0.05, Figure 6A), reduced LDH release (*P* < 0.05, Figure 6B), and decreased cardiomyocyte apoptosis (*P* < 0.05, Figure 6C, 6D). However, metformin did not show cardioprotective effect against H/R injury when the activation of NLRP3 was suppressed (P < 0.05, Figure 6A–6D).

Furthermore, we determined the expression of AMPK and the interaction of AMPK-NLRP3 *in vitro*. As shown in Figure 6E–6I, the Western blot results showed that compared with the Con group, the ratios of p-AMPK/AMPK, NLRP3/GAPDH, cleaved-caspase 1/GAPDH and IL-1β/GAPDH in the other groups were increased (*P* < 0.05). After metformin treatment, the ratio of p-AMPK/AMPK in the MET+veh group was up-regulated, and the ratios of NLRP3/GAPDH, cleaved-caspase 1/GAPDH and IL-1β/GAPDH in MET+veh group were markedly down-regulated compared with the H/R group (*P* < 0.05, Figure 6E–6I), whereas, the ratios of NLRP3/GAPDH, cleaved-caspase 1/GAPDH, and IL-1β/GAPDH in MET+nigericin group were obviously up-regulated compared to the MET+veh group (*P* < 0.05, Figure 6E–6I).

Consistent with the western blot results, the levels of IL-1β and IL-18 in all groups in the supernatant from NCVMs showed a similar trend. Compared to the Con group, the levels of IL-1β and IL-18 in the other groups were significantly enhanced (P < 0.05, Figure 6J). Metformin significantly inhibited the levels of IL-1β and IL-18 in the MET+veh group compared with H/R group (*P* < 0.05, postconditioning). Nonetheless, compared to the MET+veh group, the levels of IL-1β and IL-18 in the MET+nigericin group were significantly increased when the activation of NLRP3 was suppressed (*P* < 0.05, Figure 6J). These results suggest that metformin protects cardiomyocytes against H/R-induced injury by suppressing pyroptosis via the AMPK/NLRP3 inflammasome pathway.

## DISCUSSION

To the best of our knowledge, this is the first study that examined the regulatory effect of metformin post-conditioning on the AMPK/NLRP3 inflammasome pathway following myocardial ischemia-reperfusion injury. We found that Metformin exerts cardioprotective effect by alleviating myocardial structural lesions, suppressing myocardial apoptosis, and inhibiting inflammatory response *in vivo* and *in vitro.* In clinical practice, Metformin is a widely used oral hypoglycemic drug. Previous studies have demonstrated that metformin exerts its cardioprotective effect by regulating multiple molecular pathways. Notably, several studies have shown that metformin pre-conditioning and postconditioning could effectively reduce myocardial I/R injury by activating AMPK and its downstream signaling pathways, such as PI3K/Akt, GRP94, PGC-1α and eNOS signaling pathway [27–30].

The Langendorff heart is an *ex vivo* technique that allows the examination of cardiac contractile strength and heart rate without the complications of an intact animal or human. In this study, the degree of myocardial I/R injury was measured by analyzing myocardial infarction size, myocardial injury-related enzymes, and hemodynamics indexes. We found that metformin post-conditioning inhibits myocardial infarction size, reduces the contents of myocardial injury-related enzyme LDH, CK-MB, and cTnl, and improves hemodynamics indexes. The results from HE staining, mason staining, and Western Blot further indicated that metformin post-conditioning reduces myocardial inflammatory cell infiltration, myocardial collagen deposition, and improves myocardial histopathological changes. In addition, it suppresses the up-regulation of myocardial fibrosis-related proteins Col-I and Col-III expression levels. Our data further indicated that metformin post-conditioning could significantly inhibit the augments of myocardial apoptosis and the ratio of Bax/Bcl2 induced by myocardial I/R injury, which also provided further confirmation of the cardioprotection effect of metformin on myocardial I/R injury, suggesting that metformin reduces tissue necrosis and myocardial cell apoptosis.

With the administration of the AMPK inhibitor CC, the area of myocardial infarction and the levels of the myocardial injury-related enzyme were increased, while the hemodynamic indicators significantly deteriorated. CC is an effective, reversible, and selective AMPK inhibitor that exerts its effects by allosteric regulation and inhibiting AMPK phosphorylation activation at Thr172. After using CC to inhibit AMPK activation, the protective effect of metformin postconditioning on myocardium was abolished, and the degree of myocardial cell necrosis was increased. More importantly, this protective effect was eliminated by the AMPK inhibitor CC. Consistent with the latest research results [27–30], our data suggested that metformin postconditioning effectively reduced myocardial I/R injury by activating AMPK.

Previous studies have shown that AMPK is present in almost all tissues and cells of mammals [31]. It has an essential role in regulating energy and material metabolism and participates in various biological functions, such as cell proliferation, apoptosis, and inflammation [32]. Drugs pre-conditioning or post-conditioning exerts its cardioprotective effects against myocardial I/R injury through a variety of mechanisms, such as activating AMPK and downstream signaling pathways, regulating energy metabolism, reducing myocardial cell inflammation, inhibiting myocardial apoptosis, and regulating autophagy [33–35]. In this study, we demonstrated that metformin postconditioning could lead to the activation of AMPK and its downstream protein ACC. Meanwhile, the results from ELISA and RT-PCR indicated that metformin postconditioning effectively inhibited the up-regulation of myocardial inflammatory cytokines IL-1β, IL-18, and TNF-α induced by myocardial I/R injury. Our data confirmed that metformin has an anti-inflammatory effect on myocardial I/R injury.

Many pharmacological studies have focused on suppressing inflammation response to reduce cardiac I/R injury [36, 37]. A large number of studies have confirmed that the inflammatory response could aggravate the tissue necrosis and apoptosis in myocardial I/R injury, and the NLRP3 inflammasome has an important role in it [20, 21, 38]. NLRP3, as one of the representatives of the inflammasome nodding-like receptor (NLR) family, is currently the most extensively studied inflammatory complex protein in the body. NLRP3 can be activated by different molecules, bacteria, and viruses. After NLRP3 is activated, its N-terminal heat protein domain (PYD) binds to the ASC containing the caspase recruitment domain and aggregates caspase1 to form the NLRP3 inflammasome [39]. Under the action of autocatalysis, the precursor of caspase-1 is activated. The activated caspase-1 can then cleave the precursors of IL-1β and IL-18. Subsequently, activated IL-1β and IL-18 as main effectors have an important role in inflammation and participate in the development of myocardial I/R injury [40].

According to a recent study, adiponectin upregulation of the phosphorylation of AMPK can reduce inflammation, inhibit the activation of the NLRP3 inflammasome, while also having a beneficial role in cerebral I/R injury [41]. It is worthy of paying attention that Metformin protects against intestinal I/R injury by decreasing cell pyroptosis via the NLRP3-GSDMD axis [9]. In this study, we examined whether metformin exerts its cardioprotective effect against myocardial I/R injury by activating AMPK and inhibiting NLRP3 inflammasome activation. The expression levels of NLRP3, ASC, cleaved-caspase 1, IL-1β, and IL-18 were measured by Western Blot. We found that metformin activated AMPK, inhibited the expressions of NLRP3 and ASC, and significantly reduced the levels of cleaved caspase1, IL-1β, and IL-18, and ultimately restrained NLRP3-mediated cardiomyocyte pyroptosis. Moreover, after treatment with AMPK inhibitor CC, the expressions of NLPR3 and ASC were up-regulated, and the expressions of cleaved-caspase1, IL-1β, and IL-18 proteins increased. CC inhibited AMPK and myocardial I/R injury-induced inflammatory response and led to a significant increase in myocardial cell necrosis. Hence, our results suggest that the AMPK/NLPR3 inflammasome signaling pathway has a crucial role in resisting myocardial I/R injury-induced inflammatory response induced by metformin.

**Figure 7:**
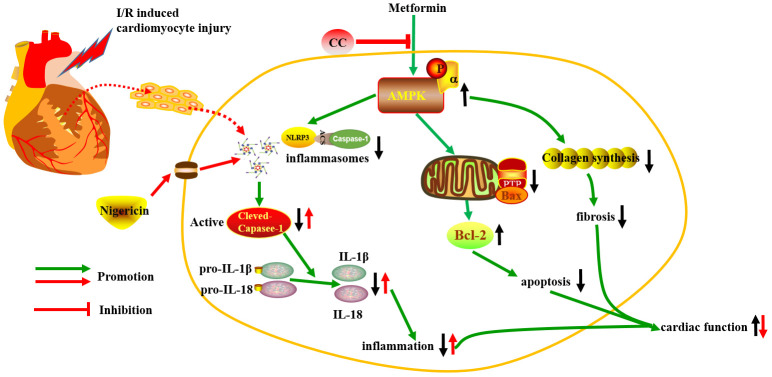
**The schematic diagram of the protective properties of Metformin against myocardial I/R injury via the AMPK/NLRP3 inflammasome pathway.**

The molecular mechanism of metformin against myocardial I/R injury is complex and involves multiple molecular pathways. As shown in Figure 7, our study suggests that metformin can prevent myocardial I/R injury and suppress inflammatory response through the activation of AMPK and its regulated NLRP3 inflammasome. Thus, AMPK and its regulated NLRP3 inflammasome can be used as potential new therapeutic targets for clinical prevention and treatment of ischemic heart disease.

## MATERIALS AND METHODS

### Animals and neonatal rat ventricle myocytes culture

The Sprague-Dawley rats (male, 180-230g) were maintained and obtained from medical laboratory animal center of Nanchang University. All the animal experiments were approved by the Institutional Animal Care and Use Committee of Nanchang University and performed in compliance with the guidelines for the Principles of Laboratory Animal Care and Use of Laboratory Animals published by NIH (NIH Publication, 8^th^ Edition, 2011).

Neonatal rat ventricle myocytes (NRVMs) were isolated from the 2-day-old Sprague–Dawley rat pups via enzymatic digestion with pancreatin and type-2 collagenase, as described before [42]. The cell suspension was preplated for 90 mins in serum-free PC-1 medium (BioWhittaker, Walkersville, MD). After removing cardiac fibroblasts, the unattached cells were NRVMs. The separated and purified NRVMs were cultured with in a mixture of 10% fetal bovine serum and antibiotics (100 μg/ml penicillin and 100 μg/ml streptomycin) in dulbecco's modified eagle medium (DMEM). The combined treatment of the experimental group was illustrated in Supplementary Figure 1 and Supplementary Figure 2. All hearts and NRVMs were randomly divided into four groups. The specific information was shown in Supplemental Materials.

### Langendorff isolated heart perfusion model

According to the previous research, the K-H solution was configured [6]. NaCl 118.0, KCl 4.8, KH_2_PO_4_ 1.2, NaHCO_3_ 25.0, MgSO_4_ 1.2, CaCl_2_ 2.5 and glucose 11.0. Adjusted the PH value to 7.35-7.45, and advance to inject into mixed gas (95% O_2_-5% CO_2_) for 30 min to fully mix the K-H liquid and the gas, keeping the circulation temperature at 37° C. Rats were intraperitoneally injected with pentobarbital sodium (50 mg/kg) for anesthesia and femoral vein heparin (500 U/kg) for heparinization, and quickly opened the chest to take the heart, placed it in 4° C K-H solution, drained the residual blood and hung on the Langendoff perfusion device. K-H buffer is continuously perfused at constant pressure (80 mmHg). A latex balloon connected to the sensor was inserted into the left ventricle through the left atrium incision, and the balloon pressure was maintained at 0-10 mmHg via injecting water. In the meantime, the hemodynamics indexes (heart rate, HR; left ventricular peak pressure, LVSP; left ventricular end diastolic pressure, LVEDP) at 30 min of equilibration (T0), 30 min (T1), 60 min (T2), 90 min (T3), and 2 h (T4) after reperfusion were monitored and recorded by using biological signal acquisition and processing system (U/4C501H Med Lab, Shanghai).

### In vitro H/R model

When NRVMs reached 80% confluence, the cells were treated with hypoxi/reoxygenation (H/R) to mimic the I/R model *in vitro*. The culture medium (90% DMEM+10% FBS) was replaced with serum-free Earle’s medium (CaCl_2_ 0.18 mmol/L, MgSO_4_·7H_2_O 0.08 mmol/L, KCl 0.05 mmol/L, NaCl 11.43 mmol/L, NaHCO_3_ 2.62 mmol/L, and NaH_2_PO_4_ 0.10 mmol/L), and then incubated for 3h in an incubator with 94% N_2_, 5% CO_2_, and 1% O_2_ at 37° C. Thereafter, earle’s medium was then replaced with normal medium, and the cells were incubated for 6 h reoxygenation in 95% air and 5% CO_2_ at 37° C.

### Myocardial infarction size measurement

At the end of reperfusion, the hearts were immediately removed from the Langendoff perfusion device. After freezing at -80° C for 5 min, 5-6 pieces of 2mm thick tissue were made with a heart cutter, and then placed these pieces in 1% triphenyltetrazolium chloride (TTC, lot number: T8877, Sigma, USA) to stain evenly. Avoid light, incubate at constant temperature for 20 min. After washing in phosphate buffered saline (PBS) buffer, 10% formalin was fixed for 24 h. The infarcted area and the non-infarcted area were stained off-white and brick red respectively. Alpha View gel image software was used to evaluate the volume of the left ventricular infarction and the total volume of left myocardium (myocardial infarction size).

### Cell viability and LDH activity assay

The cell viability of NRVMs was determined by MTT assay as describe previously [6]. Cell viability was measured with a cell counting kit-8 assay (CCK8, Shanghai Beyotime Biotechnology, Shanghai, China) according to the manufacturer’s instructions. Briefly, NRVMs were plated in 96-well plates at a density of 5,000 cells per well. After proper treatment, the wells were incubated with a mixture of 10 μL CCK-8 solution and 90 μL serum-free DMEM at room temperature for 2 h. Then, the optical density (OD) value was detected at a wavelength of 450 nm with a microplate reader.

Cardiac lactate dehydrogenase (LDH) activity was measured using a LDH assay kit (Nanjing Jiancheng Biotechnology, China), according to the manufacturer’s instructions. LDH release was expressed as units per liter (U/L).

### CK-MB and cTnI measurement

After the rat heart reperfusion for 2h, the left ventricular tissue was taken. 10% tissue homogenate was made of normal saline, the supernatant was obtained after centrifugation at 4° C, 3 000 r /min for 15 min. Creatine kinase-MB (CK-MB) and cardiac troponin I (cTnl) levels were detected using commercial kits from Nanjing Jiancheng Bioengineering Institute according to the instructions, expressed as units per liter (U/L).

### Myocardial histopathological staining

At the end of reperfusion, 1 mm^3^ of left ventricle was immediately cut and placed in 4% paraformaldehyde for fixation to prepare frozen sections. The rat heart tissue was soaked in 4% paraformaldehyde solution for more than 24h, dehydrated with ethanol and embedded in paraffin, and then sliced into 4-6μm thick slices. These slices were performed by routine hematoxylin-eosin (H and E) and mason staining as described in our previous studies [24, 25]. And the immunohistochemical (IHE) staining was performed on the next adjacent slice with a heat-induced antigen-retrieval step. All tissue sections were deparaffinized and hydrated, and endogenous peroxidase was blocked by hydrogen dioxide. The tissue sections were incubated with primary antibody against NLRP3 (1:200; Abcam, Cambridge, UK) overnight at 4° C. The negative control was the primary antibody replaced by phosphate-buffered saline (PBS). After washing with PBS, the slides were incubated using 3,3′-diaminobenzidine tetrahydrochloride (DAB, ZSGB-BIO, Beijing, China) to visualize the antigen-antibody compound. Finally, images were obtained by using a light microscope (LM, BX51, Olympus Co., Japan).

### Cell apoptosis detection

Apoptosis were detected in the myocardium and NRVMs by TUNEL staining. At 4° C, 4% paraformaldehyde was used to fix rat myocardial tissue, dehydrated with ethanol and embedded in paraffin for sectioning. And then washed with PBS (pH:7.35) for two times, the components of PBS solution are NaCl, Na2HPO4, KCl, KH2PO4. According to the operation steps of the kit, added TUNEL mixed solution and incubated at 37° C for 1h in the dark. Continue to stain the nuclei with 4,6-biamidine-2-phenylindole (DAPI), incubate for 10 min at room temperature, and rinse twice with PBS. The myocardial tissues were observed and photographed under a fluorescent microscope. The apoptotic cells showed green fluorescence in TUNEL staining, and the nucleus showed blue fluorescence. Apoptosis rate of cardiomyocytes appertained to the percentage value of the number of apoptotic cardiomyocytes and the total number of cardiomyocytes.

### Inflammatory cytokines measurement

The concentrations of inflammatory cytokines interleukin-1β (IL-1β), interleukin-18 (IL-18), and tumor necrosis factor-α (TNF-α) in cell supernatant or serum were detected using an enzyme-linked immuno sorbent assay (ELISA) kit (R&D Systems, Minneapolis, MN, USA). The specific operation steps of ELISA are strictly in accordance with the instructions of the kit, and the data was acquired by the microplate reader.

### RNA extraction and real-time PCR

The rat heart tissues were immediately taken from *in vitro* myocardial I/R injury model. According to the manufacturer’s instructions, total RNAs were extracted from rat heart tissues or NRVMs using the RNAsimple total RNA extraction kit (Tiangen, Beijing, China). The RNA concentration and purity were measured. The cDNA was synthesize using reverse transcription kit (Thermo Fisher Scientific, MA, USA) and amplified using TransStarts Green qPCR SuperMix (Transgen, Beijing, China). β-actin was used as an internal control. After cDNA synthesis, the expression levels of IL-1β, IL-18, TNF-α and the housekeeping gene β-actin were determined by real time-PCR using the FastStart Universal SYBR Green Master (Roche, Indianapolis, USA). The experiment was repeated 3 times. The primers used in the experiments were listed in Table 2 and β-actin served as an internal control.

**Table 2:** Real-time PCR primer sequences.

**Target gene**	**Sequences (5’-3’)**
IL-1β (RAT)	forword:5-TGAAAGCTCTCCACCTCAATGGAC-3
	reverse: 5-TGCAGCCATCTTTAGGAAGACACG -3
IL-18 (RAT)	forword:5-GACTCTTGCGTCAACTTCAAGG-3
	reverse: 5- CAGGCTGTCTTTTGTCAACGA-3
TNF-α (RAT)	forword: 5-CATCTTCTCAAAATTCGAGTGACAA-3
	reverse:5-TGGGAGTAGACAAGGTACAACCC-3
β-actin (RAT)	forword:5-TAAAGACCTCTATGCCAACACAGT-3
	reverse:5-CACGATGGAGGGGCCGGACTCATC-3

### Western blot analysis

Cardiomyocytes were collected and lysed by using cell lysate, including RIPA lysis buffer and protease inhibitors. After taking the supernatant and measuring the protein content by the BCA method, a protein sample was prepared. 30 μg protein sample was taken and mixed well with the buffer, and heat in the bath for 5 min. The proteins were transferred to polyvinylidene fluoride membranes (PVDF) after electrophoresis. The PVDF membranes were blocked in Tris-buffered saline with Tween-20 (TBST, 20 mM Tris, 137 mM NaCl, 0.1% Tween-20) containing 5% non-fat milk for 1 h, and then incubated with primary antibodies against Col-I (1:1000, Cell Signaling Technology, Danvers, MA, USA), Col-III (1:1000, Cell Signaling Technology, Danvers, MA, USA), pho-AMPK (1:1000, Cell Signaling Technology, Danvers, MA, USA), AMPK (1:1000, Cell Signaling Technology, Danvers, MA, USA), pho- acetyl CoA carboxylase (ACC, 1:1000, Cell Signaling Technology, Danvers, MA, USA), ACC (1:1000, Cell Signaling Technology, Danvers, MA, USA), Bax (1:1000, Abcam, Cambridge, UK), Bcl2 (1:1000, Abcam, Cambridge, UK), NLRP3 (1:1000, Abcam, Cambridge, UK), apoptosis associated speck like protein containing a CARD (ASC, 1:1000, Abcam, Cambridge, UK), Cleaved caspase-1 (1:1000, Cell Signaling Technology, Danvers, MA, USA), IL-1β(1:1000, Cell Signaling Technology, Danvers, MA, USA), IL-18 (1:500, Abcam, Cambridge, UK) and GAPDH (1:2000, BOSTER, Wuhan, China) at 4° C overnight. After being washed with TBST, the PVDF membranes were incubated with secondary antibodies for 2 h at room temperature. Subsequently, after washing, developing, grayscale scanning, the gray values of the band were analyzed by using Image J software. The protein expression was reflected by the ratio of the gray value of the target band to the gray value of GAPDH.

### Statistical analysis

Data were expressed as means ± SEM. All experimental data were analyzed by using one-way analysis of variance (ANOVA) followed by Tukey multiple comparison test. A level of *P*<0.05 was considered to be statistically significant difference.

## Supplementary Material

Supplementary Materials

Supplementary Figures
