# Supplementary material for: Metformin protects against myocardial ischemia-reperfusion injury and cell pyroptosis via AMPK/NLRP3 inflammasome pathway
Source: Aging (Albany NY). 2020 Nov 24;12(23):24270–87. doi: 10.18632/aging.202143 (PMC7762510; doi:10.18632/aging.202143)
Supplement: Supplementary Materials [file aging-12-202143-s001.pdf]

Supplementary Materials Methods

SUPPLEMENTARY EXPERIMENTAL PROTOCOLS

Sixty-four healthy rats were taken for anesthesia, and their hearts were taken directly after thoracotomy and hung on the Langendoff perfusion device to establish an isolated MIRI model. Isolated perfused rat hearts were randomly divided into four groups: Sham group, ischemia/reperfusion (I/R) group, metformin postconditioning (MET) group and MET+AMPK inhibitor (Compound C, CC) group. In the Sham group, each rat was subjected to 3h continuous perfusion. Except the Sham group, in Langendoff MIRI models were established in the other groups, each rat was subjected to 30min equilibrium period, 30min ischemia period, followed by 2h reperfusion period. In MET group and MET+CC group, the rat hearts were perfused with K-H solution respectively saturated with 50 μM metformin and 50 μM metformin combined with 10 uM Compound C for 15 min starting from the onset of reperfusion until 15 min after reperfusion, and then with plain K-H solution for 105 min. The experimental design was shown in Supplementary Figure 1.

In vitro H/R model

Neonatal rat ventricular myocytes (NRVMs) were randomly divided into four groups: Sham group, hypoxia/reoxygenation (H/R) group, metformin postconditioning+the specific vehicle-ethanol (MET+veh) group and metformin postconditioning+the specific NLRP3 activator-nigericin (MET+nigericin) group. In the Sham group, each rat was subjected to 3h continuous oxygenation. Except the Sham group, NRVMs was subjected to 3h hypoxia period, followed by 3h reoxygenation period in the other groups. In MET+veh group and MET+nigericin group, NRVMs were respectively subjected to 5 mM metformin and 5 mM metformin combined with 20 μM nigericin for 30 min starting from the onset of reoxygenation until 30 min after reoxygenation, and then with plain reoxygenation for 150 min. The experimental design was shown in Supplementary Figure 2.
