# Supplementary material for: Metformin protects against myocardial ischemia-reperfusion injury and cell pyroptosis via AMPK/NLRP3 inflammasome pathway
Source: Aging (Albany NY). 2020 Nov 24;12(23):24270–87. doi: 10.18632/aging.202143 (PMC7762510; doi:10.18632/aging.202143)
Supplement: Supplementary Figures [file aging-12-202143-s002.pdf]

Supplementary Figures

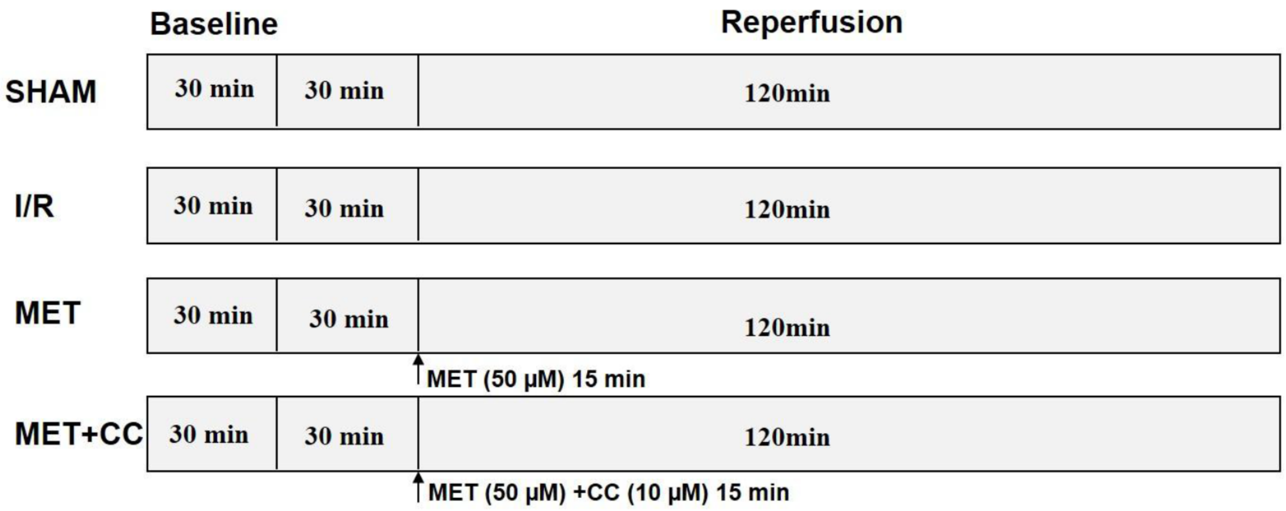

Supplementary Figure 1. Schematic illustration of the experimental protocol in Langendoff IR model. All the groups underwent the same surgical operation. (1) SHAM: rats were subjected to 3h continuous perfusion; (2) IR: rats received 30min equilibrium period, 30min ischemia period, followed by 2h reperfusion period; (3) MET: rat hearts were perfused with K-H solution saturated with 50 μM metformin for 15 min besides ischemia/reperfusion; (4) MET+CC: rat hearts were perfused with K-H solution saturated with 50 μM metformin combined with 10 uM Compound C for 15 min besides ischemia/reperfusion.

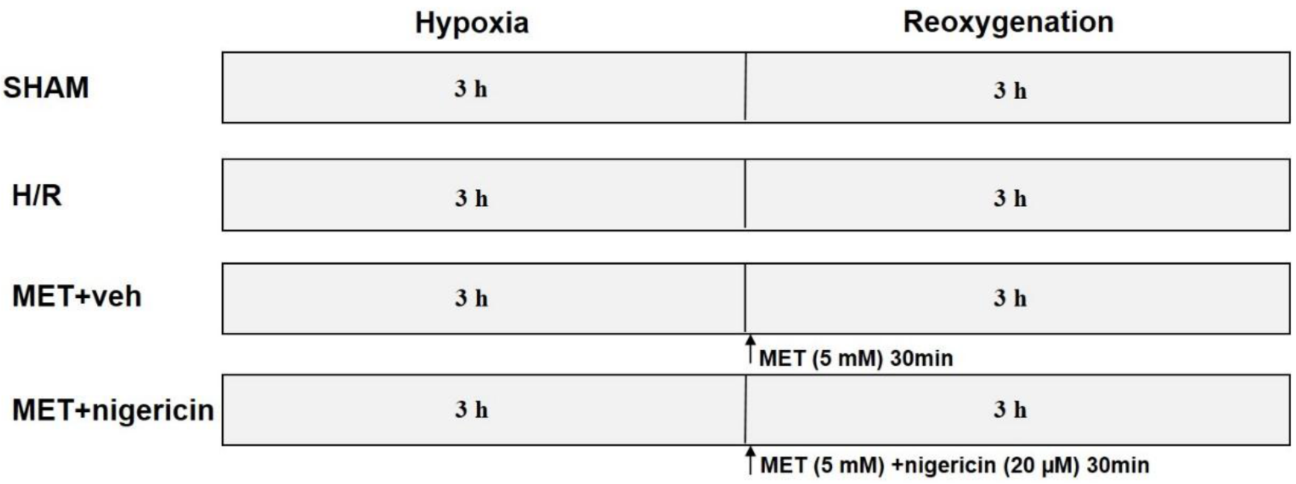

Supplementary Figure 2. Schematic illustration of the experimental protocol in vitro H/R model. Nigericin initially dissolved in ethanol and diluted with DMEM to a final concentration of 20 μmol/l. (1) SHAM: neonatal rat ventricular myocytes (NRVMs) were subjected to 3h continuous oxygenation; (2) H/R: NRVMs received 3h hypoxia period, followed by 3h reoxygenation period; (3) MET+veh: NRVMs were subjected to 5 mM metformin for 30 min besides hypoxia/reoxygenation; (4) MET+nigericin: NRVMs were subjected to 5 mM metformin combined with 20 μM nigericin for 30 min besides hypoxia/reoxygenation.
